# Persistent Human Cosavirus Infection in Lung Transplant Recipient, Italy

**DOI:** 10.3201/eid1910.130352

**Published:** 2013-10

**Authors:** Giulia Campanini, Francesca Rovida, Federica Meloni, Alessandro Cascina, Rachele Ciccocioppo, Antonio Piralla, Fausto Baldanti

**Affiliations:** Fondazione Istituto Di Ricovero e Cura a Carattere Scientifico Policlinico San Matteo, Pavia, Italy (G. Campanini, F. Rovida, A. Cascina, A. Piralla, F. Baldanti);; Università degli Studi di Pavia, Pavia (F. Meloni, R. Ciccocioppo)

**Keywords:** human cosavirus, epidemiology, persistent infection, lung transplant, transplant recipient, viruses, Italy

## Abstract

Human cosavirus is a novel picornavirus recently identified in feces from children in southern Asia. We report infection with human cosavirus in a patient in the Mediterranean area. The patient was an adult double lung transplant recipient who had chronic diarrhea associated with persistent infection with human cosavirus.

In 2008, a new virus was detected in fecal samples from children with nonpolio acute flaccid paralysis (*1*). The genome structure of the new virus was closely related to those in the family *Picornaviridae*, but phylogenetic analysis showed that it diverged from known picornaviruses. Thus, a new genus of picornaviruses was proposed: *Human Cosavirus* (common stool-associated virus; HCoSV).

Prevalence of HCoSV varies according to patient age, geographic area, and exposure to enteric viruses in general; it has been identified in fecal samples of children with acute flaccid paralysis in Pakistan and Afghanistan, in healthy children, and in an adult patient in the United Kingdom (*1*). In a study by Kapoor et al., HCoSV strains were classified into 4 species (A, B, D, E). HCoSV was also found in a child with acute diarrhea in Australia (*2*) and, subsequently, was detected in China, Thailand, and Brazil. In China, 3.2% of hospitalized and 1.6% of healthy children were HCoSV positive, and all virus strains belonged to species A (*3*); in Thailand, HCoSV was detected in an adult patient but not in children with acute diarrhea (*4*); and in Brazil, the percentage of HCoV-positive symptomatic children (3.6%) was comparable to that reported in China; however, in 2 groups of asymptomatic children from whom samples were collected at different periods, HCoSV was detected in highly divergent percentages (6.5% vs. 49.2%) (*5*). In Brazil, HCoSV was also detected in an HIV-positive adult patient with acute gastroenteritis, and ≈75% of symptomatic HCoSV-positive patients were co-infected with other gastroenteric viruses. Kapusinszky et al. recently conducted phylogenetic analysis of viral protein (VP) 3–VP1 genes, which revealed greater genetic variability of HCoSV strains, and proposed splitting species A into 24 species (A1–A24), D into 5 species (D1–D5), E into 2 species (E1–E2), and classifying F as 1 species (*6*).

We report a case of HCoSV infection in an immunocompromised woman in northern Italy and the results of retrospective HCoSV testing of 689 stored fecal samples from hospitalized patients with gastrointestinal signs and symptoms. The study was performed according to guidelines of the institutional review board of the Fondazione Istituto Di Ricovero e Cura a Carattere Scientifico Policlinico, San Matteo, Pavia, Italy, on the use of biological specimens for scientific purposes in keeping with Italian law (Article 13 D.Lgs 196/2003) and after having obtained written informed consent from the patient.

## The Study

In 2003, a 43-year-old white woman who had undergone a pulmonary lobectomy during childhood underwent bilateral lung transplantation because of severe chronic respiratory failure caused by bilateral bronchiectasis. The posttransplant period was complicated by bronchiolitis obliterans syndrome stage 1, with episodes of acute respiratory distress. The immunosuppressive regimen included tacrolimus (through level 10–15 ng/mL), low-dose steroids (0.10 mg/kg/day), and monthly courses of extracorporeal photopheresis that were initiated after a 3-month course of low-dose azythromycin (discontinued because of lack of efficacy).

In January 2012, the patient’s respiratory condition worsened and she was hospitalized. She had cough and fever (38°C), abdominal cramps, and diarrhea (6–10 bowel movements/day, liquid but not bloody). Routine fecal examination and cultures were negative for common gastrointestinal bacteria (*Mycobacteria*, *Salmonella*, *Shigella*, and *Campylobacter* spp.), parasites (protozoa and helminths), and viruses (astrovirus, norovirus, rotavirus, adenovirus). Because of the patient’s persistent gastrointestinal symptoms and a positive fecal calprotectin result, a colonoscopy was performed; it revealed inflamed cecal mucosa without ulcerative lesions. Histologic examination of colonic biopsy samples showed mild, chronic, interstitial inflammation, including eosinophils and lymphoid microaggregates. Therefore, fecal samples were examined for less common gastroenteric viral agents—rhinovirus, bocavirus, enterovirus, parechovirus, coronavirus, sapovirus, aichivirus and HCoSV—by using conventional and real-time PCR or nested reverse transcription PCR (RT-PCR) (*1*,*7*–*14*). Samples were negative for all gastrointestinal viruses searched for except for HCoSV. This virus was detected by a nested RT-PCR targeting the 5′ untranslated region (UTR) (*1*) and quantified on the basis of serial log_10_ dilution of extracted nucleic acid estimating the presence of 10^7^ RNA copies/mL in fecal samples. The 316-bp 5′ UTR amplicon was sequenced by using an ABI Prism 3100 DNA automatic sequencer (Applied Biosystems, Foster City, CA, USA), assembled by Sequencer software, version 5.0 (Gene Codes Corporation, Ann Arbor, MI, USA), and compared with available HCoSV sequences with the BLAST program (http://blast.ncbi.nlm.nih.gov). Nucleotide identity with the HCoSV species D (species D1) (GenBank accession no. FJ438908) was 93%.

Because the 5′ UTR is not sufficiently discriminatory for typing, we sequenced the highly variable region encompassing VP3–VP1 genes, obtained according to a published protocol (*6*). Phylogenetic analysis, performed with the maximum-likelihood method with parameters selected by the Model Selection tool in MEGA version 5.0 (*15*), showed that the HCoSV VP3–VP1 sequence from Italy clustered with species E strains found in Australia and Bolivia and recombinant D/E strains circulating in Nigeria. Although homology was greatest (97% nt and 81% aa identity) with a strain from Australia (GenBank accession no. FJ555055) (Figure), the hypothesis that this HCoSV strain might be a D/E recombinant strain (*6*) cannot be excluded because the recombination breakpoint between VP1 and the 2A gene was not investigated.

**Figure:**
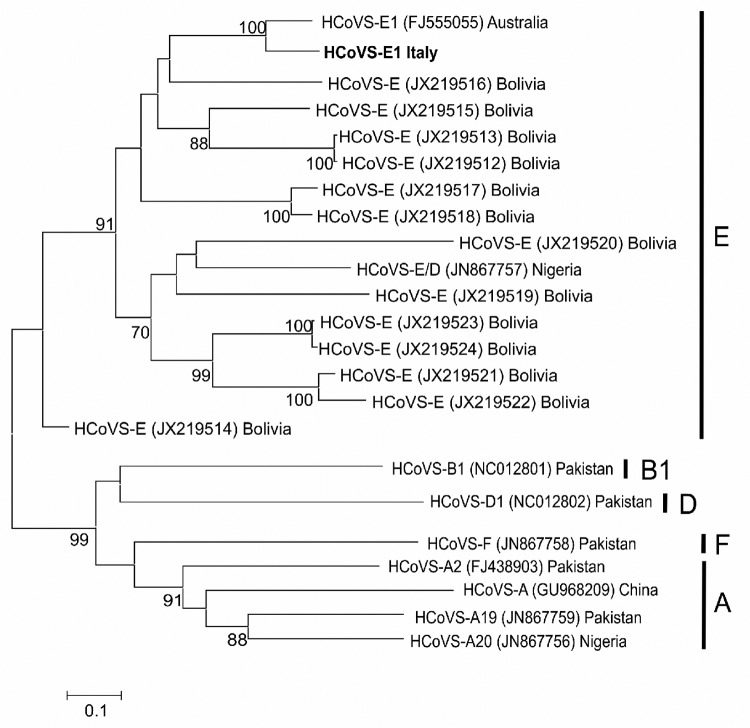
Maximum-likelihood tree based on viral protein (VP) 3–VP1 sequences. Bootstrap values were set for 1,000 repetitions and were placed over each main node of the tree. Bootstrap values <70% are not shown. The strain from the lung transplant recipient with chronic diarrhea, in Italy, is indicated in **boldface**. Reference strains from GenBank, and their accession numbers, are shown. Letters in the right-hand column indicate virus species. Scale bar indicates nucleotide substitutions per site. HCoVS, human cosavirus.

To define the circulation of HCoSV in northern Italy, we retrospectively tested 689 stored fecal samples for HCoSV positivity by using the same nested RT-PCR (*1*). The samples had been collected during April 2011–April 2012 from patients hospitalized at our hospital with gastrointestinal signs and symptoms. Of these 689 patients, 333 (48.3%) were adults (230 [69.0%] immunocompetent and 103 [31.0%] immunocompromised) and 356 (51.7%) were children (275 [77.2%] immunocompetent and 81 [22.8%] immunocompromised). Surprisingly, none of these samples were positive for HCoSV.

In the absence of specific therapy, the patient reported here underwent parenteral rehydration and nutritional supplementation; her general condition improved progressively, and her bowel movements were reduced to 2–3 per day, enabling her to be discharged within a month. However, her gastrointestinal symptoms persisted, and 3 fecal samples collected at 4, 6, and 7 months after discharge were positive for the same HCoSV strain (at a high titer of 10^5^–10^7^ virus RNA copies/mL feces) but negative for other viruses, bacteria, and parasites.

## Conclusions

We reported identification of HCoSV in the Mediterranean area. The infection was detected in 1 of 689 patients with gastrointestinal symptoms who were hospitalized and tested during the same period. Unique to this patient were the prolonged immunosuppressive therapy and the chronic gastrointestinal symptoms with persistently HCoSV-positive fecal samples. The persistence of symptoms and virus are additional new findings that might be associated with the patient’s immune impairment.

The source of infection has not been determined. However, the patient denied domestic or international travel and close contact with persons returning from travel. Because no other HCoSV infection was detected in patients hospitalized at the same institution during the same period, we hypothesize that the infection was acquired in the patient’s community before her hospitalization. The genetic similarity with E strains circulating in South America, Australia, and Central Africa suggest local acquisition of an imported infection. However, additional evidence is required before excluding the potential autochthonous circulation of HCoSV in northern Italy.
